# Use of disease modifying anti-rheumatic drugs and risk of multiple myeloma in US Veterans with rheumatoid arthritis

**DOI:** 10.1186/s41927-025-00457-3

**Published:** 2025-01-17

**Authors:** Kate Tokareva, Alexander C. Peterson, Aaron Baraff, Sarah P. Chung, Jennifer Barton, Joshua F. Baker, Bryant R. England, Ted R. Mikuls, Nicholas L. Smith, David G. Coffey, Noel S. Weiss, Namrata Singh

**Affiliations:** 1 University of Washington, Seattle, WA USA; 2 ERIC, VA Puget Sound, Seattle, WA USA; 3 Virginia Mason, Seattle, WA USA; 4 Portland VA Medical Center and OHSU, Portland, OR USA; 5 Corporal Michael J. Crescenz VA Medical Center, Philadelphia, PA USA; 6 University of Nebraska Medical Center and VA Nebraska-Western Iowa Health Care System, Omaha, NE USA; 7 Division of Myeloma, University of Miami, Miami, FL USA; 8 Department of Epidemiology, University of Washington, Seattle, WA USA; 9 Division of Rheumatology, University of Washington, Seattle, WA USA; 10 1959 NE Pacific Street, Seattle, WA 98195 USA

**Keywords:** Tumor necrosis factor inhibitors, Rheumatoid arthritis, Multiple myeloma

## Abstract

**Background:**

Biologic (b) and targeted synthetic (ts) disease-modifying anti-rheumatic drugs (DMARDs) used in the management of rheumatoid arthritis (RA) target inflammatory pathways implicated in the pathogenesis of multiple myeloma (MM). It is unknown whether use of b/tsDMARDs affects the incidence of MM.

**Methods:**

In this cohort study using Veterans Health Administration (VHA) data, we identified Veterans newly diagnosed with RA from 1/1/2002 to 12/31/2018 using diagnostic codes and medication fills. DMARD exposure was categorized as follows: conventional synthetic (cs)DMARDs; bDMARDs, which included tumor necrosis factor inhibitors (TNFi), non-TNFi; and a tsDMARD, tofacitinib. A Cox proportional hazards model with time-varying exposure was used to estimate the hazard ratio for developing MM among those who received b/tsDMARD medications relative to b/tsDMARD-naïve persons.

**Results:**

27,540 veterans with RA met eligibility criteria of whom 8322 (30%) took a b/tsDMARD during follow-up. There were 77 incident cases of MM over 192,000 person-years of follow-up. The age-adjusted incidence rate (IR) of MM among b/tsDMARD-naïve patients was 0.37 (95% CI 0.28–0.49) per 1000 person-years and 0.42 among current or former b/tsDMARD users (95% CI 0.25–0.65). Adjusting for age and other demographic characteristics, the hazard ratio for MM associated with use of b/tsDMARDs was 1.32 (95% CI 0.78, 2.26).

**Conclusion:**

In this study of Veterans with RA, the rate of MM did not differ between b/tsDMARD and csDMARD users. The relatively short duration of follow-up and few events limited our power to detect treatment-related differences in MM risk.

**Supplementary Information:**

The online version contains supplementary material available at 10.1186/s41927-025-00457-3.

## Key points

This retrospective cohort study is one of the first to look at the impact of b/tsDMARD use in a large cohort of patients with underlying RA on risk of developing multiple myeloma (MM).

We observed no statistically significant difference between b/tsDMARD use and the risk of developing MM compared to csDMARDs alone.

**Supplementary Information:**

The online version contains supplementary material available at 10.1186/s41927-025-00457-3.

## Introduction

Multiple myeloma (MM) is a hematological malignancy characterized by proliferation of clonal plasma cells that secrete monoclonal immunoglobulins in the bone marrow [1]. Several studies have suggested an excess risk of lymphoproliferative malignancies such as lymphoma, leukemias, and myeloma in people with RA [2–4]. A meta-analysis from 2014 estimated an overall relative risk of MM in people with RA to be 1.14 (95% CI: 0.97–1.33), though with substantial heterogeneity among the results of the studies [5].

Several biologic disease modifying anti-rheumatic drugs (bDMARDs) introduced in recent years target pro-inflammatory cytokines, such as tumor necrosis factor alpha (TNF-α) and interleukin-6 (IL-6), which are elevated in patients with inflammatory conditions such as RA [6, 7]. TNF-α and IL-6 are also hypothesized to promote MM development and progression [8–10]. IL-6 is a cytokine that plays a role in B-cell differentiation and functions as a growth factor for survival of MM cells. TNF-α upregulates the secretion of IL-6 and promotes adhesion of MM cells to bone marrow stromal cells, effects which may enhance MM development and progression [11]. Given the shared inflammatory pathways between RA and MM, the hypothesis of this study was that biologic agents may play a protective role in cancer pathogenesis and decrease the risk of developing MM.

While previous studies have examined the role of biologics in treating RA and the subsequent risks of developing different cancers, data on MM incidence is sparse. A nested case-control study suggested a reduced risk of developing MM in patients who received both conventional synthetic (cs) and bDMARDs compared to patients who had received csDMARDs alone [12]. However, this study cohort included patients with psoriatic arthritis and ankylosing spondyloarthritis in addition to RA, limiting inferences specific to those with RA. The objective of our study was to evaluate differences in the risk of incident MM between users of b- or targeted synthetic (ts) DMARDS in RA compared to those receiving csDMARDS in a population of United States (U.S.) Veterans with RA.

## Methods

### Data sources and study population

This cohort study included patients diagnosed with RA in any U.S. Veterans Health Administration (VHA) facility. We used electronic medical records data from the VHA’s national Corporate Data Warehouse (CDW), which included diagnoses, procedures, visits, laboratory results, medications (including outpatient, intravenous, and bar-coded medication administration (BMCA)), oncology data, and demographic information. Mortality was ascertained using the CDW’s vital status file, which incorporates information from both VA and non-VA sources, including Medicare and Social Security Administration data. The study protocol was approved by Institutional Review Boards at the University of Washington and the VA Puget Sound.

Study inclusion criteria were: (1) a diagnosis of RA, defined by the presence of two or more International Classification of Diseases (ICD) Version 9 or 10 codes for RA at least 7 days apart but no more than 365 days apart between 1/1/2002 and 12/31/2018; (2) a prescription for a csDMARD within 90 days of the first RA diagnosis (index date); (3) 18 + years of age at first RA diagnosis; and (4) one or more inpatient or outpatient visits from at least 30 days prior and up to 2 years prior to first RA diagnosis, suggesting regular VHA medical care. Patients were excluded for any of the following reasons: (1) any record of csDMARD or b/tsDMARD use prior to first RA diagnosis; (2) diagnostic codes of another inflammatory arthritis (such as psoriatic arthritis or ankylosing spondylitis) more than one year prior to first RA diagnosis (indicating uncertainty in diagnosis and/or timing); (3) diagnosis of MM before the diagnosis of RA; (4) or a history of tuberculosis or human immunodeficiency virus infection in the year prior to RA diagnosis. Similar algorithms for identifying RA have been validated in previous studies and have a positive predictive value (PPV) of greater than 90% [13].

### Exposure and outcome definitions

The csDMARDS included were methotrexate, sulfasalazine, leflunomide, and hydroxychloroquine. The bDMARDs included were tumor necrosis factor inhibitors (TNFi: adalimumab, etanercept, infliximab, certolizumab, and golimumab) and non-TNFi biologics (rituximab, abatacept, and tocilizumab), and their respective biosimilars. The tsDMARD included was tofacitinib. Additional tsDMARDs were not included in the analyses due to low numbers. For the main analysis, all medications except csDMARDs were labeled collectively as b/tsDMARDs. Once a patient initiated a b/tsDMARD they were considered exposed throughout the remainder of follow-up.

Incident MM diagnoses were ascertained by one or more ICD-9 or ICD-10 diagnoses codes from inpatient and outpatient data tables or from site and histology data from the oncology raw domain (ORD) (Supplementary Table 1). Although the VA Central Cancer Registry (VACCR) serves as the gold standard for cancer ascertainment, a substantial time lag exists between case abstraction and final inclusion within the registry [14]. As such, the ORD, which represents data abstracted at the local level by cancer registrars, mimics the quality of VACCR data and is more easily accessible to VA researchers, making it a practical resource for cancer ascertainment [14]. La et al. investigated various definitions of MM through different combinations of diagnostic codes, and found that with one diagnostic code using the cancer registry, the PPV for identifying patients with MM was 0.97 (0.95–0.98) [15].

### Covariates and statistical analysis

Steroid use was defined as the use of any formulation containing hydrocortisone, dexamethasone, methylprednisolone, or prednisone within one year prior to Time0. Rheumatic Disease Comorbidity Index (RDCI) was determined based on the presence of diagnosis codes within one year prior to Time0. Smoking status was determined based on free-text entries in the Health Factors domain of the Corporate Data Warehouse within 1 year prior to Time0. Patients who were categorized as baseline non-smokers included those that were tobacco non-users (never or quit more than one year ago) and tobacco users (current smoker, e-cigarette user, other tobacco user, quit within the past year). BMI was determined based on the median height and weight closest to but preceding Time0 up to 1 year prior to Time0.

Person-time was counted from the latter of second RA diagnosis or initiation of treatment with DMARDs (i.e. only after all inclusion criteria were met). Follow-up was censored upon date of incident multiple myeloma diagnosis, death, loss to follow-up (defined as the date of last VHA visit if no subsequent visits for 2+ years), or 12/31/2019. Exposure period for receipt of csDMARD treatment began at the initiation of that treatment and continued until loss to follow-up, initiation of a b/tsDMARD, death, or the end of the study period, depending on whichever came first. The initiation of b/tsDMARD use marked the start of exposed person-time for the use of one of these drugs, and continued until loss to follow-up, death, or the end of the study, depending on whichever came first. Incidence rates were calculated as the number of events divided by the total person-time at risk, with associated 95% confidence intervals based on the Poisson distribution.

A multivariable Cox proportional hazards model with time-varying b/tsDMARD exposure was used to estimate the hazard ratio for developing MM among those during and following the use of a b/tsDMARD relative to csDMARD treatment only. Diagnostic methods were performed to check for violations of model assumptions. Covariates to adjust for in the statistical models were selected a priori and included demographics (age, sex, and race/ethnicity). Data for race and ethnicity were missing from 8.3 to 6.4% of patients, respectively. Multiple imputations for these missing values were performed using sequential regressions against all other covariates, b/tsDMARD exposure, and the outcome. In additional sensitivity analyses, we adjusted the multivariable model for each of the following variables measured at baseline: smoking, body mass index (BMI), steroid use in the year prior, rheumatic disease comorbidity index (RDCI), and all the potential covariates combined. In another sensitivity analyses, we excluded the first 90 days as the exposed period to avoid attributing multiple myeloma diagnoses in this timeframe to the drug. MM diagnoses so close to index date might not truly be from exposure to the drug.

Because of concern that rituximab (categorized under the non-TNFi group), an anti-CD20 chimeric antibody used for the treatment of certain B-cell malignancies, could have a differential effect on cancer risk, a secondary analysis was performed that compared hazard ratios for only TNF-α inhibitors relative to csDMARD use. For this secondary analysis, b/tsDMARD exposure was further divided into periods of TNF-α inhibitor use and non-TNF-α inhibitor use. A multivariable Cox proportional hazards model with time-varying exposure was applied adjusting for demographic characteristics (age, sex, race, and ethnicity). All analyses were done using software R [16].

## Results

**Fig. 1:**
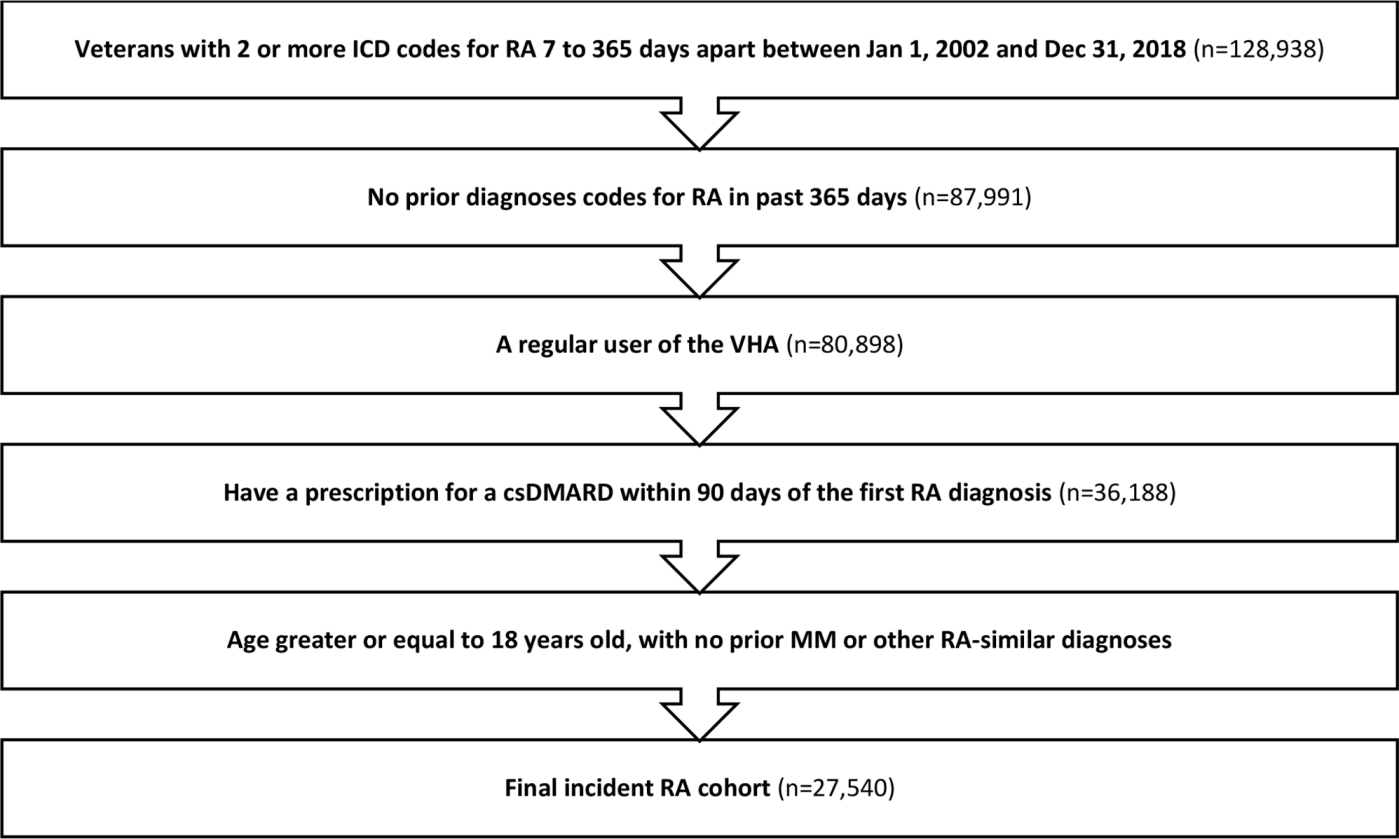
Cohort creation flowchart for patients with incident rheumatoid arthritis in the nationwide VA

A total of 27,540 patients met study eligibility criteria (Fig. 1). Of these, 19,220 (69.8%) were considered b/tsDMARD naïve, meaning they never switched to a b/tsDMARD throughout the study period, and 8320 (30.2%) initiated a b/tsDMARD at some point during the study period. The study cohort was mostly male (88.9%), White (75.5%), and non-Hispanic/Latino (89.1%), and was similar between both exposure groups (Table 1). A majority of the cohort had a positive RF or anti-CCP lab (*N* = 16,541, 60%), with higher rates among those who were ever exposed to b/tsDMARDs: 6236 (75%), and 10,305 (54%) among those never exposed.

**Table 1:** Characteristics of the person-years accrued among cohort members based on biologic disease modifying anti-rheumatic drug (bDMARD) use status

	Person-time as bDMARD naïve	Person-time of bDMARD exposure
**Demographics**	**Person-years in 1000s (%)**	
*Age (years)*		
> 65	88.4 (64.1)	27.8 (51.5)
36–65	48.2 (34.9)	25.2 (46.7)
19–35	1.3 (0.9)	1.0 (1.8)
*Sex*		
Male	123.8 (89.8)	46.6 (86.3)
*Race*		
White	104.3 (75.7)	43.2 (80)
Black	18.8 (13.7)	6.4 (11.9)
Other	3.3 (2.4)	1.4 (2.6)
Missing	11.4 (8.3)	3 (5.5)
*Ethnicity*		
Not Hispanic or Latino	123.6 (89.7)	49.4 (91.4)
Hispanic or Latino	5.8 (4.2)	2.5 (4.6)
Missing	8.4 (6.1)	2.2 (4)
*Smoking (baseline)*		
No	30 (21.8)	10.8 (20)
Yes	53.7 (38.9)	22.7 (41.9)
*BMI (baseline)*		
≤18.5	1.0 (0.7)	0.4 (0.8)
18.5–25	26 (18.9)	9.3 (17.2)
25–30	46.2 (33.5)	17.3 (32.1)
>30	45.5 (33)	19.7 (36.5)
Missing	19.2 (13.9)	7.2 (13.4)
*Cohort Entry years*		
2002–2007	69.7 (50)	26.3 (51)
2008–2013	49.2 (35)	20 (38)
2014–2019	21.3 (15)	5.7 (11)
**RA characteristics (baseline)**		
*CRP (mg/L)*		
0–3	14.5 (10.5)	5.2 (9.6)
3–30	44.9 (32.6)	19.5 (36)
30–50	5.5 (4)	2.7 (5)
50+	7.1 (5.2)	3.7 (6.9)
Missing	65.9 (47.8)	23 (42.5)
*Steroid use (prior year)*		
No	94.3 (68.4)	36.4 (67.4)
Yes	43.6 (31.6)	17.6 (32.6)
*RDCI*		
0	26.3 (19)	14.1 (26)
1	34.4 (24.9)	14.2 (26.3)
2	37.1 (26.9)	13.2 (24.4)
3	21.7 (15.8)	7.2 (13.3)
4+	18.4 (13.4)	5.4 (9.9)

*Abbreviations bDMARD* biologic disease modifying anti-rheumatic drug, *BMI* body mass index, *CRP* C-reactive protein, *RA* rheumatoid arthritis, *RDCI* rheumatic disease comorbidity index

There were 77 incident multiple myeloma cases over a total of 192,000 person years of follow-up (the median duration of follow-up was 5.8 years) (Supplemental Table 2). 55 of those events were in patients who had never taken a b/tsDMARD. After adjusting for age and other demographic characteristics, the hazard ratio for developing MM following b/tsDMARD use relative to csDMARD was 1.37 (95% CI 0.81–2.32). The additional inclusion of other demographic characteristics and clinical covariates measured at baseline, such as smoking, BMI, and steroid use within the past year, did not meaningfully change HR estimates (data not shown). In the fully adjusted model, use of b/tsDMARDs was not associated with statistically significant increase in risk of MM relative to csDMARD only use (HR 1.32, 95% CI 0.78–2.26) (Table 2).

**Table 2:** Fully adjusted multivariable Cox proportional hazards model for association between use of DMARDs and incident multiple myeloma in rheumatoid arthritis

Clinical characteristic	Adjusted hazard ratio (95% CI)	
b/tsDMARD use	1.32	(0.78, 2.26)
Age*	1.04	(1.01, 1.07)
Female	0.58	(0.20, 1.66)
Race		
Black	2.17	(1.20, 3.95)
Other	0.76	(0.10, 5.46)
Ethnicity		
Hispanic or Latino	0.7	(0.16, 3.03)
Current smoker	0.82	(0.43, 1.58)
BMI < = 18.5	0	(0.00, Inf)
BMI > 30	1.72	(0.78, 3.80)
BMI 25–30	1.7	(0.81, 3.59)
Baseline CRP 3–30	1.07	(0.50, 2.27)
Baseline CRP 30–50	2.19	(0.84, 5.76)
Baseline CRP 50+	1.61	(0.64, 4.04)
Steroid use in year prior	1.64	(1.03, 2.59)
Baseline RDCI1	0.43	(0.20, 0.96)
Baseline RDCI2	0.88	(0.46, 1.70)
Baseline RDCI3	0.91	(0.43, 1.90)
Baseline RDCI4+	1.02	(0.48, 2.15)

Model adjusted for age, sex, race, ethnicity, smoking status, BMI, CRP (in mg/l), cohort entry, steroid use in prior year, baseline RDCI

*Abbreviations bDMARD* biologic disease modifying anti-rheumatic drug, *BMI* body mass index, *CRP* C-reactive protein, *RDCI* rheumatic disease comorbidity index

*Hazards ratio reflects risk per every 1-year increase in age

In the secondary analysis comparing TNF-α inhibitor use to csDMARD only use, there were 55 MM cases diagnosed in csDMARD users who never took a b/tsDMARD, 20 events in patients after switching to a TNF-α inhibitor, and 2 MM events while on a non- TNF-α inhibitor. After adjustments for demographic characteristics, there was no statistically significant difference in MM incidence in TNF-α inhibitor users than in exclusive csDMARD users (HR 1.28, 95% CI 0.74–2.22) (Supplemental Table 3).

In sensitivity analyses excluding first 90 days after RA diagnosis/drug initiation, the results did not change considerably with the HR for biologic use being 1.21 (95% CI 0.70–2.10).

## Discussion

In this cohort study of US Veterans with RA, we evaluated the association between b/tsDMARD use and the risk of developing MM compared to csDMARDs alone and observed no statistically significant difference between the two groups.

In an earlier study of this question (12), Calip et al. identified 287 cases of multiple myeloma during 2009–2015 among patients with RA, psoriatic arthritis, or ankylosing spondylitis enrolled in health care plans contributing data to the Truven Health MarketScan Databases. The investigators compared patients using various forms of anti-rheumatic medications to other enrolled patients with one of these three conditions who had not been diagnosed with multiple myeloma. 13.9% of the patients with myeloma had received two or more prescriptions for a bDMARD, compared to 15.1% of controls (odds ratio 0.84, 95% confidence interval 0.57–1.26). Differences in study design and population contribute to challenges in making direct comparisons between the results of this study compared to ours, but do not demonstrate evidence of a statistically significant risk difference between b/tsDMARDs and csDMARDs.

MM is a malignancy of plasma cells, a type of B cell, so the results of studies of other B cell malignancies (such as most lymphomas) have potential relevance. To date, several studies among patients with a history of RA have observed little difference between lymphoma cases and controls regarding prior use of b/tsDMARDs [17–19].

This retrospective cohort study is one of the first to look at the impact of b/tsDMARD use on risk of developing MM in a large cohort of patients with underlying RA. Despite this being a very large study using health record data, the number of events was small, limiting power for the primary analysis and limiting our ability to evaluate individual therapies. Similarly, because of the relatively small number of women in the VA system and the higher incidence of MM in men, we could not examine possible differences by sex regarding the impact of these medications. Another potential limitation resulted from not having information on indicators of RA severity as of the time of initiation of a b/tsDMARD. If patients who switched to a b/tsDMARD had more severe RA compared to those who stayed on a csDMARD, and if RA severity is also positively associated with the occurrence of MM, then our estimated relative risk for MM among b/tsDMARD users could be inaccurately elevated. Finally, although MM is a disease that in many patients plausibly takes decades to develop [20], the median follow-up in our study was only 5.8 years. In the study by Calip et al. the majority of bDMARD users also had received the drugs for a short period of time (less than 2.5 years). To accurately evaluate the long-term impact of b/tsDMARD use on the incidence of MM, studies with a longer period of follow-up are needed. The independent variable in our study was defined as ever exposure to b- or tsDMARD. There is a chance that someone was exposed to these agents for only a small duration.

## Conclusion

In our nationwide cohort of U.S. Veterans with incident RA, rates of MM did not differ between b/tsDMARD users and non-users. While these data are reassuring against a large increase in risk, longer follow-up of this and other large cohorts of RA patients will be needed before any conclusions can be reached regarding the impact use of these medications on MM occurrence.

## Electronic supplementary material

Below is the link to the electronic supplementary material.

Supplementary Material 1

Supplementary Material 2

Supplementary Material 3

## Data Availability

The deidentified datasets used and/or analyzed during the current study are available from the corresponding author on reasonable request.

## Abbreviations

RA: Rheumatoid Arthritis
VHA: Veterans Health Administration
MM: Multiple myeloma
csDMARD: conventional synthetic disease modifying anti-rheumatic drugs
bDMARD: biologic disease modifying anti-rheumatic drugs
TNFi: Tumor necrosis factor inhibitor
CI: Confidence interval
CDW: Corporate Data Warehouse
ORD: Oncology raw domain
