# Supplementary material for: Use of disease modifying anti-rheumatic drugs and risk of multiple myeloma in US Veterans with rheumatoid arthritis
Source: BMC Rheumatol. 2025 Jan 17;9:7. doi: 10.1186/s41927-025-00457-3 (PMC11740324; doi:10.1186/s41927-025-00457-3)
Supplement: Supplementary file 1 — Supplementary Material 1 [file 41927_2025_457_MOESM1_ESM.docx]

**Supplemental Table 1**: Incidence rates for multiple myeloma by DMARD status

|  | Events (n=77) | Person-Years/1000 | Age-adjusted Incidence Rate (95% CI) * | Unadjusted Hazard Ratio (95% CI) |
| --- | --- | --- | --- | --- |
| b/tsDMARD naïve | 55 | 138 | 0.37 (0.28-0.49) | Reference |
| b/tsDMARD treated | 22 | 54 | 0.42 (0.25-0.65) | 1.04 (0.63-1.74) |
| *Median follow-up was 5.8 years | | | | |

CI: confidence interval; bDMARD: biologic disease modifying anti-rheumatic drug; ts: targeted synthetic.

* Per 1000 person-years
