# Supplementary material for: Use of disease modifying anti-rheumatic drugs and risk of multiple myeloma in US Veterans with rheumatoid arthritis
Source: BMC Rheumatol. 2025 Jan 17;9:7. doi: 10.1186/s41927-025-00457-3 (PMC11740324; doi:10.1186/s41927-025-00457-3)
Supplement: Supplementary file 2 — Supplementary Material 2 [file 41927_2025_457_MOESM2_ESM.docx]

**Supplemental Table 2**: Multivariable Cox proportional hazards model for association between use of tumor necrosis factor inhibitors (TNFi) and incident multiple myeloma

| Clinical characteristic | Adjusted hazard ratio (95% CI) |
| --- | --- |
| csDMARD  TNFi | Reference  1.28 (0.74-2.22) |
| Age* | 1.04 (1.02-1.07) |
| Female | 0.57 (0.20-1.61) |
| Race | |
| White  Black  Other | Reference  2.17 (1.20-3.93)  0.74 (0.10-5.37) |
| Hispanic Ethnicity | 0.77 (0.18-3.28) |
| Abbreviations: b-/tsDMARD- biologic or targeted synthetic disease modifying anti-rheumatic drug; CI: confidence interval  *Hazards ratio reflects risk per every 1-year increase in age  Model adjusted for age, gender, race, and ethnicity | |
