# Supplementary material for: Use of disease modifying anti-rheumatic drugs and risk of multiple myeloma in US Veterans with rheumatoid arthritis
Source: BMC Rheumatol. 2025 Jan 17;9:7. doi: 10.1186/s41927-025-00457-3 (PMC11740324; doi:10.1186/s41927-025-00457-3)
Supplement: Supplementary file 3 — Supplementary Material 3 [file 41927_2025_457_MOESM3_ESM.docx]

| Supplementary Table 3: ICD9/10 codes for RA and MM | |
| --- | --- |
| RA | MM |
| \| 714.0 \| \| --- \| \| 714.1 \| \| 714.2 \| \| 714.4 \| \| 714.81 \| \| 714.89 \| \| 714.9 \| \| M05.00-M06.9 \| | \| 203.0 \| \| --- \| \| 203.00 \| \| 203.00 \| \| 203.01 \| \| 203.02 \| \| C90.00 \| \| C90.01 \| \| C90.02 \| |
